# Hospital Construction

**Published:** 1896-09-12

**Authors:** 


### HOSPITAL CONSTRUCTION.

### PLENUM VENTILATION AND HOSPITAL PLANS.

A correspondent writes: "Only those who study what is possible with the plenum system, properly applied, can realise the practicability of such an arrangement." This is the dictum of Mr. William Henman, F.R.I.B.A., in a letter to the editor of the *Builder*, published in the number of that journal dated August 8th, 1896. The "arrangement" referred to is to erect hospital wards of one storey only, placed side by side, and lighted by continuous lantern lights. But why "lantern lights"? In such wards, without side windows, the only views which can be presented to the patients is one of the sky, which can be seen but very imperfectly through lantern lights. How much better it would be to make the entire roof of glass, and compel the patients to remain in their beds, on their backs, that they may obtain the full benefit of the "view." The side walls need not be more than six feet high, while the peak in the centre may rise to twelve feet. With wards eighteen feet wide, lying side by side, and separated by nine-inch walls, which would be quite strong enough to carry the light glazed roof, very close packing could be secured at a minimum of cost. Moreover, something might be done by means of the "plenum" to utilize the exhalations of the sick instead of turning them loose into the outer air as a dangerous waste product. Construct beneath the glazed troughs which are to serve as wards a series of caves through which the foul air, expelled by the "plenum" system, is to be forced. Then plant these caves with edible mushrooms, and treat them as is done in the mushroom caves of Paris. The offensive nitrogenous exhalations of the sick will furnish precisely the nutriment which is most eagerly assimilated by the fungi. The caves will be kept moist and warm, the foul air will be purified, and can be taken up by the plenum fans and used over again, and many other advantages of the plan will become apparent when it is carefully considered. Of course all this might be done by an exhaust system also; but as the only architect who is ever likely to try such experiments appears to be entirely devoted to the "plenum" system it must be done by the latter method or not at all.

We are much impressed with Mr. Henman's statement that "architectural effect is of slight importance to the welfare of patients, and of ease in administration," it seems to indicate that the hospitals of the future may be planned by the proprietors of patent fans, who have given some attention to the construction of conservatories, and that a new branch of industry is on the verge of springing from the old stock. We must not be too sanguine, however; there is much wisdom in the remark of the old Scotchman: "And so the devil's dead; ah, weel, I'd no bury him until he began to smell a wee strong like."

⁂ We gladly publish the above letter from a correspondent. holding up to ridicule, as it does, a proposal which has recently been made in regard to the facilities for hospital construction placed at the disposal of architects by the plenum system of ventilation. The proposal is, in fact, that, instead of erecting pavilions, "it might be better to spread out the wards on one storey only, placed side by side, and lighted by continuous lantern lights," and we do not wonder at our correspondent being moved to mirth, and led to write in a vein of ridicule regarding it. One is indeed tempted to follow his example, and one might go even further in the same line, were it not for the gravity of the matter in question. When one finds a man of Mr. Henman's position so far led astray, by a fairly logical deduction from the data on which he starts, as to make such a ridiculous suggestion, we may be quite sure that there is something wrong in the data themselves, and Mr. Henman's letter to the *Builder* may be taken as a *reductio ad absurdum* of the claims of the plenum system of ventilation as applied to hospitals. Not that we would say that plenum ventilation, or mechanical ventilation, for we see no necessity to tie ourselves to one system, should never be made use of. It has its uses, but in regard to these uses Mr. Henman has got hold of quite the wrong end of the stick. If there is plenty of land nothing is easier than to build a pavilion hospital all on one level, as is being done at Halifax, and although the distance to be travelled looks greater on the plan, the labour of distribution is actually less than where stairs and lifts are necessary. It is not, however, in one-storey hospitals that difficulties arise in ventilation, or that the plenum system will ever find its full utility, but rather in those cases where, for want of space, wards have been piled one above the other in great blocks. Wherever wards are on one level there can be no difficulty in arranging separate and independent ventilation for each, but where several storeys of wards are superposed, and great enclosed staircases pass from floor to floor, and lift-shafts form great flues, carrying air from one level to another, the isolation of the wards is not so simple, and here, if anywhere, mechanical ventilation will be useful, especially in the upper floors. But it must always be remembered that, however useful mechanical ventilation may be in such cases, its use is to overcome evils forced upon the architect by the necessities of the position and the climatic conditions, and not as a system good in itself. To fall back when such conditions are absent on a purely mechanical method of obtaining what exists in plenty all around, viz., fresh air, is, we think, a retrograde step, and is one which ignores the advantages placed at our disposal by modern science and mechanical skill. While Mr. Henman would supply his patients with piped fresh (?) air, forced through miles of culverts by his pumping engine, the pavilion system would break up vast hospitals into small blocks, each deriving its supply of air fresh from the outside; and, admitting that there are days when natural ventilation tends to fail, the modern facilities which are given us by electricity for the distribution of power would enable us, while using the pavilion plan, not only to overcome the difficulty of transport from ward to ward, but to set in action local fans in the different wards whenever the atmospheric effects were such as to interfere with natural ventilation. We by no means say that mechanical ventilation should never be used, but we do say that this latest proposal shows a very wrong conception of its proper scope, especially in a temperate climate, like that of the British Isles, where mechanical ventilation is an unnecessary and costly luxury, exclusive dependence on which makes against, and not for, the improved hygienic condition of hospital wards.

